# Vaccinations and Infections Are Associated With Unrelated Antibody Titers: An Analysis From the German Birth Cohort Study LISA

**DOI:** 10.3389/fped.2019.00254

**Published:** 2019-06-25

**Authors:** Mahrrouz Caputo, Heike Raupach-Rosin, André Karch, Michael Borte, Irina Lehmann, Uwe Gerd Liebert, Marie Standl, Joachim Heinrich, Rafael T. Mikolajczyk

**Affiliations:** ^1^Department of Epidemiology, Helmholtz Centre for Infection Research, Brunswick, Germany; ^2^PhD Programme “Epidemiology,” Brunswick, Germany; ^3^German Centre for Infection Research (DZIF), Site Brunschwick-Hannover, Brunswick, Germany; ^4^Institute for Epidemiology and Social Medicine, University of Münster, Münster, Germany; ^5^Children's Hospital, Municipal Hospital St. Georg Leipzig, Academic Teaching Hospital of the University of Leipzig, Leipzig, Germany; ^6^Department of Environmental Immunology, Core Facility Studies, Helmholtz Centre for Environmental Research- UFZ, Leipzig, Germany; ^7^Charitè – Universitätsmedizin Berlin and Berlin Institute of Health (BIH), Berlin, Germany; ^8^Institute of Virology, Leipzig University, Leipzig, Germany; ^9^Helmholtz Zentrum München- German Research Center for Environmental Health, Institute of Epidemiology, Munich, Germany; ^10^Institute and Outpatient Clinic for Occupational, Social and Environmental Medicine, University Hospital of Munich, Ludwig-Maximilians-Universität München, Munich, Germany; ^11^Institute for Medical Epidemiology, Biometry, and Informatics (IMEBI), Medical Faculty of the Martin-Luther-University Halle-Wittenberg, Halle (Saale), Germany

**Keywords:** humoral response, vaccinations, infectious diseases, non-specific effects, immune response

## Abstract

The evidence for non-specific effects (NSE) of vaccinations on all-cause morbidity and mortality among children is growing. However, our understanding of the underlying mechanisms is still limited. One hypothesis is that NSE are mediated by antibody titers. We used data of 2,123 children from the population-based birth cohort study LISA conducted in Germany to explore whether routine childhood vaccinations and the individual infection history in the first 2 years of life are associated with unrelated antibody titers. We selected 19 exposures (infections and vaccinations) and investigated their association with levels of 12 IgG antibody titers at the age of 2 years. Based on univariable analyses (ANOVA), we identified 21 crude associations between exposures and titers (*p* < 0.05), while 11 (95%-CI: 6, 17) spurious associations were expected due to multiple testing. In exploratory multivariable analyses, we observed associations between seven investigated IgG titers and 10 exposures; either administered vaccines [e.g., higher anti-hRSV IgG titer in BCG-vaccinated children (regression-coefficient in standard-deviation-units: 0.38; 95%-CI: 0.12, 0.65)] or infections [e.g., higher anti-measles IgG titer in children with reported chickenpox (0.44; 95%-CI: 0.08, 0.80)]. Our results indicate the existence of associations between immunogenic exposures and unrelated antibody titers. Further studies investigating the underlying immunological mechanisms are required.

## Introduction

Epidemiological studies in low-income countries suggested that immunization with certain vaccines can have non-specific effects (NSE) on all-cause morbidity and mortality among children (1, 2). Studies on NSE of vaccines were mostly conducted in low-income countries, but some findings were also replicated in high-income settings (3–5). One of the potential mechanisms discussed in the context of NSE is a non-specific modulation of the immune system. This has been supported by several studies showing that the uptake of live attenuated vaccines, such as Bacillus Calmette-Guérin (BCG) against tuberculosis and the vaccine against measles, has been associated with decreased morbidity and mortality to unrelated pathogens (6–8). In contrast to the observed beneficial NSE of live attenuated vaccines, DTP (an inactivated vaccine) seemed to induce negative NSE (8–10). The underlying immunological mechanisms of NSE are still unknown. Findings of animal studies and investigations on non-specific immunomodulation in adults indicated that infections affect the immune response also in a non-specific manner and alter the reaction toward unrelated subsequent infections (7). Furthermore, different factors, e.g., geographical location or immunization during pregnancy, can have NSE on the infants' vaccine response (11). However, it remains unclear which pathways are involved in the modulation of the immune system; in particular, little is known regarding strengthening or weakening of antibody response to unrelated pathogens (12). Preferably, the effects should be studied in children, during the development of the immune system. However, assessing such effects on the antibody responses is complicated by the fact that not only vaccinations, but also infections during infancy and mothers' exposures during pregnancy can potentially modulate antibody titers (11). Further, the time of exposure as well as accumulation of exposures might influence the immune response (13–17). The objective of our study was to investigate whether routine childhood vaccinations and the individual history of infections in the first 2 years of life as well as maternal exposures during pregnancy are associated with the modulation of antibody titer levels against unrelated pathogens at the age of 2 years.

## Materials and Methods

### Study Population

**Figure 1:**
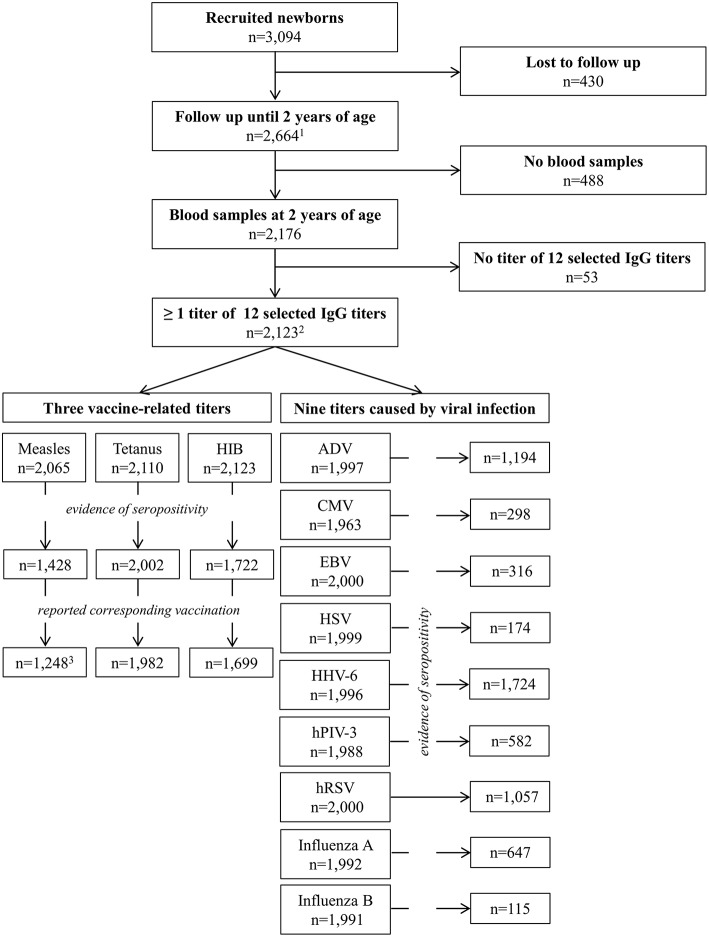
Flow Chart of included children by investigated IgG titers. (1) Of these 2,661 completed the 2-years-questionnaire. (2) Of some children the serum was insufficient for detection of certain IgG titers. (3) One child with corresponding measles infection excluded.

We used data from the birth cohort “*Influence of Life-style factors on Development of the Immune System and Allergies in East and West Germany* (LISA),” which was described in detail elsewhere (18, 19); 3,094 healthy term newborns from four regions of Germany (Munich, Wesel, Leipzig, and Bad Honnef) were recruited between December 1997 and January 1999. Of these, 2,664 (86.1%) were followed up until the age of 2 years (Figure 1). Their parents were asked to complete questionnaires every 6 months during the first 2 years of life. At the age of 2 years, blood samples were obtained from children and 12 antibody titers were measured. The LISA study was carried out in accordance with the recommendations of all relevant guidelines. The protocol was approved by the responsible ethic committees of all study centers (Bavarian Medical Council, University of Leipzig, Medical Council of North Rhine-Westphalia). All subjects gave written informed consent in accordance with the Declaration of Helsinki.

### Exposure Variables—Infections and Vaccinations of the Child and Maternal Exposures During Pregnancy

Based on self-reported data, we assessed exposure variables including vaccinations and infections of the child and maternal exposures during pregnancy. Information on experienced infections and obtained vaccinations of the children was collected retrospectively, approximately every 6 months using self-administered questionnaires. Parents were asked to consult the vaccination card when filling out the questionnaire and to record physician-diagnosed infections. To assess the influence of timing of the corresponding exposure, we classified each exposure of the child into four mutually exclusive categories: only in the first year, only in the second year, in both years, and neither in the first nor in the second year of life (reference group). We also combined different respiratory infections (pneumonia, bronchitis, obstructive or spastic bronchitis, and otitis media) into one variable since the risk of misclassification between these infections was assumed to be high. Similarly, vaccinations administered typically at the same time or as combinations were summarized into one variable [measles, mumps, and rubella as MMR; diphtheria, tetanus, pertussis, polio, and *Haemophilus influenzae type b* (HIB) as DTPPHIB]. The German standing committee on vaccinations (STIKO) provides recommendations about which vaccines should be given at which age (20, 21). The recommended vaccinations are not obligatory. Whether the vaccination is carried out or not is the responsibility of the parents. Data about socio-demographic and -economic factors, as well as maternal exposures during pregnancy including smoking, alcohol consumption, vaccinations, and infections of the mother, were also obtained through questionnaires. The participating mothers could indicate whether they have experienced any infection and whether they have received a vaccination during pregnancy (response options “yes,” “no”). In addition, they could specify the type of infection/vaccination in a free-text field. Due to sample size limitations, we could not analyze different infections and vaccinations during pregnancy separately. We only included the dichotomized variable summarizing all reported infectious diseases [“infections during pregnancy” (yes/no)], and similarly all received vaccinations during pregnancy [“vaccinations during pregnancy” (yes/no)].

In total, we considered 19 different exposures including four routine childhood vaccinations (MMR, DTPPHIB, hepatitis B, BCG), 13 infectious diseases (any respiratory infection, pseudocroup, pertussis, roseola, chickenpox, diarrhea, thrush, urinary tract infection, worm infection, scarlet fever, mumps, measles, and rubella) as well as two maternal exposures during pregnancy (any vaccination and any infection during pregnancy).

### Outcome Variables—IgG Antibody Titers

Blood samples of the participating children in the LISA study were obtained at the age of 2 years. Three antibody titers related mainly to vaccinations (IgG against measles, tetanus, and HIB) and nine titers predominately related to viral infections (adenovirus (ADV), cytomegalovirus (CMV), Epstein-Barr virus (EBV), human herpesvirus 6 (HHV-6), human parainfluenza virus 3 (hPIV-3), human respiratory syncytial virus (hRSV), herpes simplex virus (HSV), and influenza A and B) were measured (Figure 1). Antibody titers were determined using routine enzyme-linked immunosorbent assays (ELISAs) or indirect immunofluorescence at the Institute for Virology of Leipzig University. In some cases, the serum was insufficient for the detection of certain IgG titer.

### Statistical Analysis

#### Inclusion Criteria

For our analysis, we only included children with at least one of the twelve selected IgG titers measured. To ensure that the analyzed titers against measles, HIB, and tetanus were related to vaccinations, we included only seropositive cases for whom parents reported the corresponding vaccination and excluded children for whom parents reported the corresponding infection. We defined NSE as elevated/decreased titers of antibodies unrelated to the given infection/vaccination. To investigate only NSE, established associations between related infection/vaccination and the corresponding IgG titer were not assessed (such as the association between infections with roseola and HHV6 antibody titer, or infections with measles and measles antibody titer).

#### Transformation of IgG Antibody Titers

Since for some IgG titers dilution series were reported, we considered the reciprocal values. The antibody titers were transformed using the Box-Cox method (bcskew0 function in Stata Version 12) (22) and subsequently z-transformed to ensure comparability of estimates across different analyses.

#### Bivariate and Multivariable Association Analyses

First, we tested the association between each single of the 19 investigated exposures (four vaccinations, 13 infections, and two exposures during pregnancy) and each of the 12 transformed seropositive IgG titers separately using one-way analysis of variance (ANOVA). We did not apply classic correction methods for multiple testing, which are associated with several limitations in the context of exploratory analyses (23). Instead, we assessed whether more associations below a pre-defined *p*-value cut-off were observed than expected at random. For this purpose, we conducted a random permutation among the categories of the exposures and subsequently tested their association with IgG titers. This procedure was repeated 1,000 times. The median number of significant results (*p*-value < 0.05) and the corresponding empirical 95% confidence intervals (95%-CI) were obtained and compared to the number of actually observed significant associations. Second, we included all exposure variables with *p* < 0.25 from the bivariate analyses in a multivariable linear regression model for each of the 12 IgG titers in an exploratory analysis. We applied for each regression model a backward variable selection process (*p* > 0.05 for removal of variables from the model based on Wald test). Sex of the child, maternal age at birth (as continuous variable), education level of the parents [highest level received by either parent: low- without school-leaving certificate or lower secondary school certificate (<10 years); intermediate- secondary school certificate (= 10 years); high- general qualification for university entrance (>10 years)] and region of recruitment (four levels: Munich, Leipzig, Bad Honnef and Wesel) were forced into the model for adjustment. In addition, if an association with smoking and alcohol consumption during pregnancy was observed within the bivariate analysis (*p* < 0.25), the model was also adjusted for these variables. Initially, only cases with complete data were considered. In order to investigate potential bias due to missing values in exposure variables, we performed a sensitivity analysis using the missing indicator method (recoding missing values into an additional category “missing”). All analyses were performed with Stata for Windows, version 12 (StataCorp, College Station, Texas).

## Results

### Description of the Study Population, Exposures, and Analyzed Titers

Two-thirds of the participants (*n* = 2,123) had at least one available IgG antibody titer at the age of 2 years out of the 12 selected (Table 1). There were only minor differences in baseline characteristics between those with available antibody titers and those without. Depending on the type of vaccination, data were missing for 13–50% of the children (Table 2). Taking missing values into account, 10.6% reported that their children received a BCG vaccination in the first 2 years of age (rarely reported vaccination); 51.0% of the children received a vaccination against MMR while 59.4% a vaccination against hepatitis B, and 86.4% of the children were vaccinated against DTPPHIB (often reported vaccination) (Table 2). In case of the MMR vaccine, we categorized all those who did not report all three MMR components (*n* = 326) as “missing,” since we could not distinguish between reporting errors and decision to vaccinate only selected vaccines. In contrast to self-reported vaccinations, the amount of missing data for infectious diseases was much lower (up to 6.6%). Infection rates displayed three patterns as well: rarely reported diseases (<5%) were e.g., scarlet fever or mumps; common childhood infections (thrush or roseola) were reported by up to 30%; and more frequent diseases such as diarrhea and respiratory infections were reported by over 50%. Several infections were only recorded in the second year of life (e.g., scarlet fever). 13.3% of the expectant mothers experienced infections, and 15.4% were vaccinated during pregnancy. The number of children with seropositive IgG titers varied across all 12 analyzed titers (Figure 1 and Supplementary Table S1). Of the 1,428 children with evidence of anti-measles seropositivity, 85.7% (*n* = 1,249) reported to have ever been vaccinated against measles. The remaining children were excluded from the analysis. Additionally, one child, for whom measles infection and vaccination was reported, was excluded; thus, we studied only vaccine-induced measles antibodies. For anti-tetanus and anti-HIB seropositive children, 99% reported to have received the corresponding vaccination. About 80% were seropositive for HHV-6. Between 15 and 60% of the children were seropositive for IgG antibodies against ADV, EBV, hPIV-3, hRSV, CMV, and influenza A. In <10% of the children, seropositive IgG titers against HSV and influenza B were observed.

**Table 1:** Baseline characteristics of participants in the LISA study.

**Characteristics**	**All participants *N* = 3,094**	**Participants with an available IgG titer *N* = 2,123^a^**
Sex of child, % *(n/N)*		
Female	48.8 (1,510/3,094)	47.5 (1,009/2,123)
Male	51.2 (1,584/3,094)	52.5 (1,114/2,123)
Year of birth, % *(n/N)*		
1997	1.3 (41/3,094)	1.5 (31/2,123)
1998	92.0 (2,845/3,094)	92.0 (1,953/2,123)
1999	6.7 (208/3,094)	6.6 (139/2,123)
Median age of the mother at birth, y *(Q1;Q3)*		
	31 (28/34)	31 (29/34)
Education of parents, % *(n/N)*^b^		
Low	6.2 (188/3,057)	4.2 (88/2,102)
Intermediate	29.6 (906/3,057)	27.4 (576/2,102)
High	64.2 (1,963/3,057)	68.4 (1,438/2,102)
Study location, % *(n/N)*		
Munich	47.3 (1,464/3,094)	48.5 (1,029/2,123)
Leipzig	31.5 (976/3,094)	30.5 (647/2,123)
Bad Honnef	9.9 (306/3,094)	11.2 (238/2,123)
Wesel	11.3 (348/3,094)	9.8 (209/2,123)
Provided questionnaires, % *(n/N)*		
Birth questionnaire	100.0 (3,094/3,094)	100.0 (2,123/2,123)
Half-year questionnaire *(0–5 months of age)*	91.3 (2,825/3,094)	99.1 (2,104/2,123)
1-year questionnaire *(6–11 months of age)*	88.4 (2,734/3,094)	97.9 (2,078/2,123)
One-and-half year questionnaire *(12–18 months of age)*	87.5 (2,707/3,094)	98.2 (2,085/2,123)
2-years questionnaire *(19–24 months of age)*	86.0 (2,661/3,094)	99.5 (2,113/2,123)

*Q1, first quartile (25%); Q3, third quartile (75%)*.

a *Children included with at least one available IgG titer of 12 selected antibody titers*.

b *Low, without school-leaving certificate or lower secondary school certificate (<10 years); intermediate, secondary school certificate (= 10 years); high, general qualification for university entrance (>10 years)*.

**Table 2:** Exposure to vaccinations and infections during first 2 years of life as well as during pregnancy of participants in the LISA study.

**Reported infections**	**Only first year^a^ % (*n*)**	**Only second year^a^ % (*n*)**	**Both years^a^ % (*n*)**	**No infection^a^ % (*n*)**	**Missing^a^ % (*n*)**
Respiratory infection^b^	5.8 (123)	16.8 (356)	69.7 (1,480)	4.9 (104)	2.8 (60)
Pseudocroup	2.0 (43)	7.4 (157)	1.5 (32)	83.2 (1,767)	5.8 (124)
Pertussis	0.3 (7)	0.2 (4)	0.1 (2)	93.6 (1,988)	5.8 (122)
Roseola	13.2 (280)	14.2 (302)	4.7 (100)	61.2 (1,300)	6.6 (141)
Chickenpox	5.4 (115)	11.3 (239)	0.7 (15)	77.1 (1,637)	5.5 (117)
Diarrhea with or without fever	11.4 (242)	29.2 (620)	19.0 (404)	35.1 (745)	5.3 (112)
Thrush in the mouth or diaper area	19.5 (413)	10.2 (217)	8.6 (183)	56.6 (1,201)	5.1 (109)
Urinary tract infection^c^	–	2.7 (57)	–	94.2 (2,000)	3.1 (66)
Worm infection	0	0.3 (6)	0	94.5 (2,007)	5.2 (110)
Scarlet fever^c^	–	2.4 (51)	–	94.8 (2,012)	2.8 (60)
Mumps^d^	–	0.1 (3)	–	98.9 (2,100)	0.9 (20)
Measles^d^	–	0.1 (2)	–	99.0 (2,101)	0.9 (20)
Rubella^d^	–	0.5 (11)	–	98.5 (2,092)	0.9 (20)
**Obtained vaccinations against**	**Only first year**^a^ **% (*****n*****)**	**Only second year**^a^ **% (*****n*****)**	**Both years**^a^ **% (*****n*****)**	**No vaccination**^a^ **% (*****n*****)**	**Missing % (*****n*****)**
Tuberculosis (BCG)	8.9 (189)	0.5 (10)	1.2 (25)	39.1 (831)	50.3 (1,068)
DTPPHIB^e^	12.4 (264)	1.2 (26)	72.8 (1,545)	0.7 (15)	12.9 (273)
Hepatitis B	11.2 (238)	5.0 (106)	43.2 (916)	14.9 (317)	25.7 (546)
MMR^f^	1.5 (32)	45.4 (964)	4.1 (86)	16.2 (344)	32.8 (697)^g^
**Maternal exposures^h^**	**Ever during pregnancy % (*****n*****)**			**Never during pregnancy % (*****n*****)**	**Missing % (*****n*****)**
Vaccination		15.4 (326)		84.4 (1,791)	0.3 (6)
Infection		13.3 (283)		86.4 (1,834)	0.3 (6)

a *Only first year, only children vaccinated/infected within the first year of age; Only second year, only children vaccinated/infected within the second year of age; Both years, only children vaccinated/infected during both, first and second year of age; No infection/vaccination, no vaccination/infection in the first 2 years of life; Missing, missing values*.

b *Includes pneumonia, otitis media, obstruct or spastic bronchitis, respiratory diseases and bronchitis*.

c *Only asked in children aged 12–24 months*.

d *Only asked in children aged 19–24 months*.

e *Includes vaccinations against diphtheria, tetanus, polio, pertussis, and Haemophilus influenzae type b (HIB)*.

f *Includes vaccinations against mumps, measles, and rubella*.

g Children with incomplete data on vaccination against all three, mumps, measles and rubella, were classified as “Missing.”

h *Dichotomous-categorized variables*.

### Bivariate Association Analyses

Overall, we identified 21 significant associations between exposures and studied antibody titers (Table 3). Based on permutation, 11 (95%-CI: 6, 17) spurious associations could be expected at a significance level of 5%. When considering missing values as additional category in the bivariate analysis, we observed 24 significant associations (Table 4), 16 associations were observed in both analyses.

**Table 3:** Bivariate associations between infections, vaccinations, exposures during pregnancy, and IgG antibody titers of participants in the LISA study (*p*-values for *F*-test from ANOVA).

**Exposures**	**Antibody titer^a^**											
	**CMV**	**ADV**	**EBV**	**HHV-6**	**hRSV**	**hPIV-3**	**HSV**	**Influenza A**	**Influenza B**	**Measles^h^**	**HIB^h^**	**Tetanus^h^**
**Reported infections^b^**												
Respiratory infection^c^	0.686	0.705	0.313	0.914	0.585^i^	0.309^i^	0.091	0.135^i^	0.552^i^	0.222	n.a.	**0.040**
Pseudocroup	0.256	0.808	0.192	0.324	0.849^i^	0.381^i^	0.947	0.622^i^	0.271^i^	0.587	**0.026**	0.735
Pertussis	0.611	0.732	0.370	0.341	0.624	0.418	0.947	0.503	–	0.206	0.113	0.251
Roseola	0.254	0.658	0.263	n.a.	0.620	0.976	0.312	0.435	0.656	0.827	0.566	0.798
Chickenpox	0.732	0.138	0.347	0.818	0.175	0.522	0.361	0.575	0.641	**0.042**	0.416	0.358
Diarrhea	0.937	0.250	0.278	0.245	0.675	0.763	0.497	0.280	0.711	0.253	0.601	0.554
Thrush in the mouth or diaper area	0.219	0.817	0.526	**0.011**	0.697	0.383	0.414	0.091	0.742	0.191	0.932	0.154
Urinary tract infection^d^	0.792	0.864	0.352	0.913	0.427	0.863	**0.001**	0.356	0.481	0.308	0.386	0.908
Worm infection	–	0.667	0.919	0.782	0.658	0.273	0.448	0.238	–	0.892	0.712	0.813
Scarlet fever^d^	0.946	0.447	0.350	0.345	0.520	0.592	0.970	0.774	0.548	0.206	0.506	0.231
Measles^e^	–	**0.020**	–	0.422	0.187	0.271	–	–	–	n.a.	0.963	0.068
Mumps^e^	0.940	0.362	0.082	0.565	0.181	–	0.819	0.408	–	0.054	0.946	0.726
Rubella^e^	**0.012**	0.828	0.760	0.623	0.925	**0.042**	0.299	0.785	–	0.538	0.979	0.200
**Obtained vaccination against^b^**												
Tuberculosis	**0.044**	0.231	0.605	**0.020**	**0.001**	0.538	0.914	0.052	0.948	0.054	0.509	0.051
DTPPHIB^f^	0.511	0.541	0.935	0.727	0.834	0.121	0.442	0.194	0.988	**0.003**	*n.a*.	*n.a*.
Hepatitis B	0.144	0.555	0.799	0.431	0.851	0.555	0.863	0.656	0.204	**0.045**	**0.003**	** <0.001**
MMR	0.229	0.548	0.425	0.402	0.240	0.505	0.937	0.542	0.902	n.a.	**0.020**	**0.032**
**Exposures during pregnancy^g^**												
Vaccinations^g^	0.554	0.672	0.509	**0.005**	0.220	0.356	0.347	0.283	0.901	0.060	**0.022**	0.435
Infections^g^	0.557	0.998	**0.046**	0.803	**0.027**	0.274	0.362	0.833	0.323	0.397	0.190	0.543

*ADV, adenovirus; CMV, cytomegalovirus; EBV, Epstein-Barr virus; HHV-6, human herpesvirus 6; HIB, Haemophilus influenzae type b; hPIV-3, human parainfluenza virus 3; hRSV, human respiratory syncytial virus; HSV, herpes simplex virus; MMR, combine vaccination against mumps measles and rubella; –, No p-value calculated, since no observation in this category; n.a., not analyzed, since only non-specific effects are analyzed; bold values indicates p <0.05*.

a *Normalized and standardized antibody titers of children; reported p-value by ANOVA*.

b *Four categories for exposures: only in first year, only in second year, in both years, and in neither of the 2 years of age (reference group); respectively for reported infectious disease or obtained vaccination*.

c *Includes pneumonia, otitis media, obstruct or spastic bronchitis, respiratory diseases, and bronchitis*.

d *Only asked in children aged 12–24 months*.

e *Only asked in children aged 19–24 months*.

f *Includes vaccination against diphtheria, tetanus, polio, pertussis, and HIB*.

g *Vaccination/infection ever during pregnancy vs. vaccination/ infection never during pregnancy (dichotomized coded)*.

h *Only IgG titers of children with corresponding vaccination; children with corresponding infection were excluded*.

i *While the specific viruses can cause respiratory infections and lead to a pseudocroup, they are not their single, or most common cause, and therefore, the exposure were kept in the analysis (none displayed a significant association with the respective IgG titer)*.

**Table 4:** Bivariate associations between infections, vaccinations, exposures during pregnancy including missing values, and IgG antibody titers of participants in the LISA study (*p*-values for *F*-test from ANOVA).

**Exposures**	**Antibody titer^a^**											
	**CMV**	**ADV**	**EBV**	**HHV-6**	**hRSV**	**hPIV-3**	**HSV**	**Influenza A**	**Influenza B**	**Measles^h^**	**HIB^h^**	**Tetanus^h^**
**Reported infections^b^**												
Respiratory infection^c^	0.696	0.801	0.339	0.419	0.190^i^	0.405^i^	0.154	0.179^i^	0.656^i^	0.321	n.a.	**0.020**
Pseudocroup	0.158	0.914	0.279	0.202	0.664^i^	0.340^i^	0.818	0.774i	0.441^i^	0.497	**0.013**	0.055
Pertussis	0.368	0.866	0.558	0.415	0.273	0.165	0.175	0.669	0.578	0.079	**0.038**	**0.012**
Roseola	0.355	0.792	0.277	*n.a*.	0.283	0.524	0.245	0.568	0.794	0.547	0.623	0.354
Chickenpox	0.315	0.219	0.462	0.836	0.113	0.151	0.121	0.736	0.734	**0.029**	0.397	0.142
Diarrhea	0.787	0.338	0.404	0.171	0.252	0.615	0.529	0.423	0.824	0.391	0.397	**0.036**
Thrush in the mouth or diaper area	0.058	0.693	0.686	**0.014**	0.442	0.512	0.438	0.158	0.757	0.201	0.399	**0.012**
Urinary tract infection^d^	0.937	0.817	0.409	0.981	0.727	0.739	**0.001**	0.192	0.667	0.173	0.459	0.194
Worm infection^d^	0.706	0.886	0.925	0.411	0.414	0.350	**0.011**	0.496	0.880	0.321	0.344	0.099
Scarlet fever^d^	0.230	0.642	0.572	0.628	0.693	0.862	0.369	0.508	0.592	0.353	0.256	0.116
Measles^e^	0.505	0.062	0.389	0.705	0.188	0.502	0.766	0.187	–	n.a.	0.613	0.115
Mumps^e^	0.799	0.615	0.153	0.825	0.184	0.683	0.933	0.298	–	**0.024**	0.612	0.571
Rubella^e^	**0.035**	0.960	0.660	0.868	0.449	0.117	0.569	0.565	–	0.225	0.828	0.300
**Obtained vaccination against^b^**												
Tuberculosis	0.108	0.290	0.751	**0.010**	**0.001**	0.590	0.978	**0.033**	0.447	**0.023**	0.622	0.107
DTPPHIB^f^	0.568	0.620	0.698	0.667	0.854	0.079	0.647	0.279	0.981	**0.005**	n.a.	n.a.
Hepatitis B	0.129	0.671	0.463	0.555	0.783	0.661	0.931	0.799	0.166	**0.037**	**0.008**	** <0.001**
MMR	0.299	0.709	0.526	0.559	0.313	0.669	0.933	0.367	0.078	n.a.	**0.041**	0.065
**Exposures during pregnancy^g^**												
Vaccinations	0.668	0.806	0.509	**0.017**	0.220	0.651	0.347	0.536	0.901	0.144	0.072	0.698
Infections	0.669	0.944	**0.026**	0.800	**0.027**	0.501	0.583	0.953	0.323	0.464	0.389	0.630

*ADV, adenovirus; CMV, cytomegalovirus; EBV, Epstein-Barr virus; HHV-6, human herpesvirus 6; HIB, Haemophilus influenzae type b; hPIV-3, human parainfluenza virus 3; hRSV, human respiratory syncytial virus; HSV, herpes simplex virus; MMR, combined vaccination against mumps, measles, and rubella; –, No p-value calculated, since no observation in this category; n.a., not available, since only non-specific effects are analyzed; bold values indicates p <0.05*.

a *Normalized and standardized antibody titers of children; reported p-value by ANOVA*.

b *Five categories for exposures: only in first year, only in second year, in both years, missing values, and in neither of the 2 years of age (reference group); respectively for reported infectious disease or obtained vaccination*.

c *Includes pneumonia, otitis media, obstruct or spastic bronchitis, respiratory diseases, and bronchitis*.

d *Only asked in children aged 12–24 months*.

e *Only asked in children aged 19–24 months*.

f *Includes vaccination against diphtheria, tetanus, polio, pertussis, and HIB*.

g *Vaccination/infection ever during pregnancy vs. vaccination/infection never during pregnancy (dichotomized coded)*.

h *Only IgG titers of children with corresponding vaccination; children with corresponding infection were excluded*.

i *While the specific viruses can cause respiratory infections and lead to a pseudocroup, they are not their single or most common cause, and therefore, the exposure were kept in the analysis (none displayed a significant association with the respective IgG titer)*.

### Multivariable Analyses of Association

Multivariable regression models with evidence for an association between exposure variables and the respective titer after backward selection are displayed in Table 5; each outcome titer was analyzed in a separate model. The reported regression coefficients are presented in units of standard deviation of the standard normal distribution; they are thus comparable across the models in terms of magnitude. Because of the different number of incomplete cases for specific variables, the sample size differs across the models. For five IgG antibody titers (ADV, CMV, EBV, influenza A, and influenza B) no significant associations remained after backwards selection. For seven antibody titers, there were in total 10 significant associations with exposure variables. Chickenpox during the first year of life (i.e., prior to measles vaccination) was associated with an increased measles IgG titer compared to children who did not experience chickenpox during the first 2 years. Thrush during the second year of life was associated with an elevated HHV-6 IgG titer. Vaccinations against hepatitis B and tuberculosis during the first year of life were associated with decreased tetanus IgG titer level when compared to children who received neither vaccination. Vaccination against MMR was associated with an increased anti-HIB IgG titer. Since the number of individuals in the actual reference group “children with no DTPPHIB vaccination” was too low, we considered the category “DTPPHIB vaccination in both years” as the reference group. Accordingly, children vaccinated against DTPPHIB only during the first year had a lower IgG measles vaccine-related titer compared to children vaccinated against DTPPHIB during both years. BCG vaccine in the first year of life was associated with an increased hRSV IgG titer. Infections during pregnancy were associated with reduced hRSV IgG titers. For two antibody titers we detected significant associations (hPIV-3 and rubella during the year of life; HSV and urinary tract infection in the second year of life) with a low number of observations in the respective category (*n* = 2).

**Table 5:** Adjusted associations between vaccinations, infections, and exposures during pregnancy and IgG titers of participants in the LISA study^*^.

**Antibody titer (*n*)^a^**	**Exposure (*n*)**	**Time^b^**	**Regression coefficient^c^**	**95%-CI**	***p*-value**
**Measles**^**d,e**^ (599)	Chickenpox (31)	1.	0.441	0.082, 0.799	0.016
	Chickenpox (65)	2.	0.218	−0.035, 0.472	0.092
	Chickenpox (4)	1. and 2.	−0.920	−1.887, 0.046	0.062
	Vaccination against DTPPHIB^f^ (85)	1.	−0.367	−0.593, −0.141	0.002
	Vaccination against DTPPHIB^f^ (1)	2.	−0.441	−2.363, 1.481	0.652
**Tetanus**^**d,e**^ (842)	Vaccination against hepatitis B (131)	1.	−0.323	−0.550, 0.097	0.005
	Vaccination against hepatitis B (68)	2.	0.065	−0.198, 0.328	0.629
	Vaccination against hepatitis B (414)	1. and 2.	0.167	0.005, 0.339	0.057
	Vaccination against tuberculosis (139)	1.	−0.282	−0.478, −0.087	0.005
	Vaccination against tuberculosis (5)	2.	−0.708	−1.559, 0.143	0.103
	Vaccination against tuberculosis (12)	1. and 2.	−0.250	−0.815, 0.314	0.385
**HIB**^**d,e**^ (968)	Vaccination against MMR (23)	1.	0.259	−0.194, 0.713	0.262
	Vaccination against MMR (700)	2.	0.202	0.038, 0.367	0.016
	Vaccination against MMR (61)	1. and 2.	−0.017	−0.314, 0.280	0.909
**HHV-6** (816)	Thrush (169)	1.	0.020	−0.153, 0.193	0.818
	Thrush (92)	2.	0.359	0.140, 0.579	0.001
	Thrush (71)	1. and 2.	0.037	−0.209, 0.283	0.770
**hRSV** (483)	Vaccination against tuberculosis (83)	1.	0.382	0.120, 0.645	0.004
	Vaccination against tuberculosis (1)	2.	−1.429	−3.389, 0.532	0.153
	Vaccination against tuberculosis (8)	1. and 2.	−0.054	−0.773, 0.666	0.883
	Infection (65)	During pregnancy vs. not^g^	−0.279	−0.545, −0.014	0.039

*HHV-6, human herpesvirus 6; HIB: Haemophilus influenzae type b; hRSV, human respiratory syncytial virus; MMR, combine vaccination against mumps measles and rubella*.

* *For two antibodies (hPIV-3 and HSV) the final model had <10 observations in the exposed group; these significant results are not presented*.

a *Multivariable linear regression, backward selection of independent variables of normalized and standardized IgG antibody titers; only antibody titers were considered with evidence of seropositivity*.

b *1., exposure only in first year of age; 2., exposure only in second year of age; 1. and 2., exposure only in first 2 years of age; reference group: children with no corresponding exposure in the first 2 years of age*.

c *Regression coefficients are presented in units of standard deviation of the standard normal distribution; adjusted for sex of child, age of the mother at birth, education level of the parents, and study center*.

d *Only IgG titers of vaccinated children; children with corresponding infection were excluded*.

e *In addition adjusted for smoking during pregnancy*.

f *DTPPHIB: vaccination against diphtheria, tetanus, pertussis, HIB and polio; no estimator for “no vaccination” exist, since no measles antibody titer could be detected, accordingly the category “vaccination in both years (1. and 2.)” was selected as reference group*.

g *Binary coded*.

In the sensitivity analysis with missing indicator method, we observed 12 significant associations involving nine of the 12 IgG titers (Supplementary Table S2). Apart from three associations (anti-hRSV titer and infection during pregnancy; anti-tetanus titer and vaccination with BCG; anti-HIB titer and vaccination against MMR), all findings from the primary analysis could be reproduced. Moreover, we found additional significant associations for the IgG titers against ADV, EBV, HIB, influenza A, and HHV-6.

## Discussion

Using data from a large birth cohort study, we analyzed the association between maternal exposures during pregnancy and infections/vaccinations of the child in the first 2 years of life on the one side and 12 IgG antibody titers measured at the age of 2 years on the other side.

Modulation of IgG antibody titer levels could be a potential mechanism of the NSE of vaccinations described in previous research. The number of identified significant associations between specific exposures and antibody titers exceeded the number expected due to multiple testing only. Some of our findings were in line with previous studies, whereas others have not been described before. In the following, we compare our findings with the literature.

In our study, BCG vaccination, which was mostly given during the first year of life, was associated with a significant increase of hRSV IgG titer compared to children not vaccinated against BCG. Recent reviews concluded that live attenuated vaccines such as BCG, have beneficial NSE on the neonatal mortality (8, 10). Furthermore, it has been reported that BCG was associated with a lower risk of acute lower respiratory infections in children in a study based on Demographic and Health Surveys from 19 developing countries (27), which was confirmed in an epidemiological study conducted in a developed country (4). Findings in a study in Guinea-Bissau (28) indicated that BCG vaccination has a specific protective effect against acute lower respiratory infections caused by hRSV, with the effect being most marked in girls. The beneficial NSE of BCG vaccination was already used to successfully improve the T_H_1 cell immunity against RSV by recombinant BCG strains expressing RSV-proteins in mice (29). The authors assumed that BCG expressing RSV antigen is a potential candidate for a new vaccine to prevent hRSV infection during infancy.

In contrast to the observed beneficial NSE on hRSV titer, BCG-vaccinated children in the first year of life had a lower tetanus IgG antibody titer compared to non-BCG immunized children in our study. However, this effect disappeared in the analysis including missing data, thus suggesting that the initially observed association was possibly an artifact. In agreement with this observation, Ota et al. (12) showed no significant differences in the antibody response toward tetanus toxoid in BCG-vaccinated newborns compared to not vaccinated ones in a randomized trial (12). This finding is also in line with a study on Australian infants as well as with the recent investigation of Nissen et al. (24) in Danish neonates (24, 30).

We also observed a non-specific association between MMR vaccination at the age of 1 year and elevated anti-HIB IgG titer at the age of two. However, this association disappears in our sensitivity analysis suggesting that this effect could also be an artifact. For DTP vaccine, a recent review concluded that it can possibly have negative effects on mortality (10) and a follow-up analysis argued that the evidence is even stronger (9). We were not able to examine NSE of DTP: in comparison to non-vaccinated children since only 0.7% of the children were not vaccinated against DTPPHIB (Table 2). In addition, as we have grouped vaccines, which were typically administrated at the same time, we could not assess the effect of DTP alone. Instead, we considered “vaccination in both years (1. and 2.)” as reference category. We observed lower measles antibody titres in children vaccinated against DTPPHIB in the first year only compared to children vaccinated in both years. This could suggest that effects differ dependent on whether DTPPHIB vaccines are given only before MMR vaccine or before and after. de Bree et al. discussed that the sequence of vaccinations may play a role, and that the most recently obtained vaccination affects the immune response non-specifically (8).

Furthermore, we observed higher vaccination-related anti-measles IgG titers in children who experienced chickenpox during the first year of life, while chickenpox at older age did not affect the anti-measles titer levels. The observed effect could be explained by either a specific sensitive phase of the development of immunity or the fact that exposure to chickenpox preceding vaccination can be more important than exposure following vaccination. The latter explanation would suggest that modulation of the initial immune response is the mechanism rather than enhancement of already existing IgG levels due to chickenpox infection.

In previous studies, the effects of infections during pregnancy on the immune response of infants were primarily investigated for severe infections, like human immunodeficiency virus (HIV) or malaria (11). While we did not observe any NSE for vaccinations during pregnancy, we observed an association between infections during pregnancy and a decreased hRSV IgG antibody titer in the multivariable analysis. However, the initially observed association was no longer significant in the analysis including missing data. Accordingly, this finding could also be an artifact. This part of the analysis was only exploring an unspecific hypothesis regarding recent exposure to vaccination or infections—not the lifetime exposure of the mother, which could also result in passive immunity in the newborn.

The underlying immunological mechanisms of NSE have not yet been clarified. So far, two main explanations have been discussed concerning the NSE of certain vaccines; one is related to the T-cell-mediated cross-reactivity (heterologous immunity) relating the observed NSE of vaccinations to the encoded antigens, which induce a cross-reaction with other pathogens (6, 7, 26, 31). This mechanism can include modulation of antibody titers. The second explanation assumes that the beneficial effects might base on epigenetic reprogramming of innate immune cells, termed the *trained immunity*. Evidence is growing that adaptive features of the innate immune system protect against infections independently from specific T and B cells of the adaptive immune system and seem to exhibit an immunological memory (6, 25, 32–34). These two concepts explain how a pathogen or vaccination can induce non-specific immune response toward an unrelated pathogen, which might lead to the either beneficial or adverse NSE on subsequent infections. While our findings seem to support the first explanation, innate immunity could also be involved in increased antibody titers via e.g., increased maturation or activation of antigen presenting cells. There can be different explanations of this phenomenon, but the available data do not allow more in-depth analyses. However, as Gil et al. (7) discussed, epidemiological studies suggest that the sequence of vaccinations or infections may have an impact on the immune response toward subsequent unrelated infections.

### Strengths and Limitations

Since infections and vaccinations were self-reported retrospectively, misclassification is possible. However, parents were asked to consult the children's vaccination cards. In addition, we included only infections reported as diagnosed by the physician, to reduce the risk of misclassification. Still, there is the possibility of misclassification of infections with similar symptoms (all respiratory infections were reclassified into a single variable as they bore the highest risk of misclassification). On the other side, infections for which parents did not seek medical consultation were not considered. In some cases, sub-clinical or unreported infections could affect vaccine-related titers for the same diseases (more likely for HIB, than for measles for example). Some epidemiological studies indicated different NSE of the BCG-vaccine depending on early or late administration during the neonatal period [review in (8)], however, we could not assess this issue due to insufficient data on timing of BCG vaccination.

Since not all participating parents answered each question regarding infections and obtained vaccinations, this resulted in low numbers of observations for some analyses if seropositivity was rare as well. In these cases, we had low statistical power and might have missed true associations, e.g., for the antibody titers hPIV-3 and HSV with low number of observations in the respective groups (*n* = 2). To ensure that no systematic bias was caused by removal of incomplete data, we performed additional bivariate as well as multivariable analyses including missing values as a separate category. Since the results of the complete case analyses were mostly reproduced in the missing indicator method analyses, it can be suggested that the findings were largely robust.

For investigating NSE of exposure on the immune response of already experienced infections, we only considered seropositive children for each IgG titer. It is possible, however, that children with low immune response just below the threshold were excluded even though an immune response existed. In addition, for some children an analysis for certain IgG titers was not possible, since the amount of blood in the sample was insufficient. To obtain comparable values, we transformed and standardized the IgG antibody titers; however, obtaining normal distribution was not possible for all of them. We adjusted for general demographic data, e.g., sex of the child and education of parents as proxy for socioeconomic status which are common confounders of health-related processes. Additionally, we controlled for alcohol consumption during pregnancy and smoking—both variables were linked to immunological markers in past research. However, we did not consider the effect of breastfeeding as a potentially immunomodulatory variable (35).

The LISA study is one of the largest population-based cohort studies in children with information on immune status. We were able to investigate the association of vaccinations, infections, and exposures during pregnancy on the immune response in a single dataset. In contrast to previous epidemiological studies on NSE, we used a very broad approach and assessed the impact of diverse co-variables to find out whether previously unreported associations exist. To our knowledge, we were the first investigating the NSE of the individual infection history of children on the humoral immune response. Some of our findings in this exploratory study were in line with already published data. Nevertheless, to our knowledge some observations have not been discussed in the literature before. While the number of observed significant associations was larger than the number expected, some associations were due to chance. For the correction of possible false positive findings due to multiple testing, we did not consider strategies to adjust *p*-values, such as Bonferroni correction; instead we performed a permutation test. Bonferroni correction focusses on identifying truly significant associations but is overly conservative when applied to medium to large numbers of tests and could also increasing Type II (i.e., false negative) error (36). The purpose of using permutation tests was the question of assessing whether there are overall true statistical associations in the data, without pointing out which of the associations are true. In general, permutation-based tests have become widely accepted in studied involving multiple statistical testing (37). In contrast, the multivariable analysis was purely exploratory and should only inform further research.

## Conclusion

In conclusion, our results indicate that for some routine childhood vaccinations and the individual infection history of a child there are associations with selective modulation of the humoral immune response in early childhood. This might be linked to the previously described changes in morbidity and mortality rates associated with NSE of certain vaccines. However, there is still need for further studies to better understand underlying immunological mechanisms involved in NSE of vaccines and the effects of the individual infection history. Long-term consequences of a better understanding of NSE could be the adjustment of current vaccination strategies in order to support the optimal maturation of the immune system.

## Ethics Statement

The LISA study was carried out in accordance with the recommendations of all relevant guidelines, of all study centers (Bavarian Medical Council, University of Leipzig, Medical Council of North Rhine-Westphalia). The protocol was approved by responsible ethic committees of all study centers (Bavarian Medical Council, University of Leipzig, Medical Council of North Rhine-Westphalia). All subjects gave written informed consent in accordance with the Declaration of Helsinki.

## Author Contributions

MC conducted the statistical analysis, drafted, and revised the manuscript supervised by RM. RM and AK guided the statistical analysis and supported the interpretation of the results. HR-R has contributed to the writing. MB, IL, and JH provided comments on the manuscript and supported the interpretation of the results. MS provided relevant recommendation for the statistical analysis. UL conducted the serological analysis and provided comments on the manuscript and supported the interpretation of results. All authors have read and approved the final manuscript.

### Conflict of Interest Statement

The authors declare that the research was conducted in the absence of any commercial or financial relationships that could be construed as a potential conflict of interest.

## References

[1] Aaby P Kollmann TR Benn CS. Nonspecific effects of neonatal and infant vaccination: public-health, immunological and conceptual challenges. Nat Immunol. (2014) 15:895–9. 10.1038/ni.2961 25232810

[2] Sankoh O Welaga P Debpuur C Zandoh C Gyaase S Poma MA. The non-specific effects of vaccines and other childhood interventions: the contribution of INDEPTH Health and Demographic Surveillance Systems. Int J Epidemiol. (2014) 43:645–53. 10.1093/ije/dyu101 24920644 PMC4052142

[3] Sørup S Benn CS Poulsen A Krause TG Aaby P Ravn H. Live vaccine against measles, mumps, and rubella and the risk of hospital admissions for nontargeted infections. JAMA. (2014) 311:826–35. 10.1001/jama.2014.470 24570246

[4] de Castro MJ Pardo-Seco J Martinón-Torres F. Nonspecific (Heterologous) protection of neonatal BCG vaccination against hospitalization due to respiratory infection and sepsis. Clin Infect Dis. (2015) 60:1611–9. 10.1093/cid/civ144 25725054

[5] Sørup S Benn CS Stensballe LG Aaby P Ravn H. Measles–mumps–rubella vaccination and respiratory syncytial virus-associated hospital contact. Vaccine. (2015) 33:237–45. 10.1016/j.vaccine.2014.07.110 25446818 PMC4270443

[6] Benn CS Netea MG Selin LK Aaby P. A small jab – a big effect: nonspecific immunomodulation by vaccines. Trends Immunol. (2013) 34:431–9. 10.1016/j.it.2013.04.004 23680130

[7] Gil A Kenney LL Mishra R Watkin LB Aslan N Selin LK. Vaccination and heterologous immunity: educating the immune system. Trans R Soc Trop Med Hyg. (2015) 109:62–9. 10.1093/trstmh/tru198 25573110 PMC4351360

[8] de Bree LCJ Koeken VACM Joosten LAB Aaby P Benn CS van Crevel R. Non-specific effects of vaccines: current evidence and potential implications. Semin Immunol. (2018) 39:35–43. 10.1016/j.smim.2018.06.002 30007489

[9] Aaby P Ravn H Fisker AB Rodrigues A Benn CS. Is diphtheria-tetanus-pertussis (DTP) associated with increased female mortality? A meta-analysis testing the hypotheses of sex-differential non-specific effects of DTP vaccine. Trans R Soc Trop Med Hyg. (2016) 110:570–81. 10.1093/trstmh/trw073 27856947 PMC5155548

[10] Higgins JPT Soares-Weiser K López-López JA Kakourou A Chaplin K Christensen H. Association of BCG, DTP, and measles containing vaccines with childhood mortality: systematic review. BMJ. (2016) 355:i5170. 10.1136/bmj.i5170 27737834 PMC5063034

[11] Kampmann B Jones CE. Factors influencing innate immunity and vaccine responses in infancy. Philos Trans R Soc B Biol Sci. (2015) 370:20140148. 10.1098/rstb.2014.0148 25964459 PMC4527392

[12] Ota MOC Vekemans J Schlegel-Haueter SE Fielding K Sanneh M Kidd M. Influence of *Mycobacterium bovis* bacillus calmette-guerin on antibody and cytokine responses to human neonatal vaccination. J Immunol. (2002) 168:919–25. 10.4049/jimmunol.168.2.919 11777990

[13] Ohuma EO Okiro EA Ochola R Sande CJ Cane PA Medley GF. The natural history of respiratory syncytial virus in a birth cohort: the influence of age and previous infection on reinfection and disease. Am J Epidemiol. (2012) 176:794–802. 10.1093/aje/kws257 23059788 PMC3481264

[14] Jacobson JS Goldstein IF Canfield SM Ashby-Thompson M Husain SA Chew GL. Early respiratory infections and asthma among New York City Head Start children. J Asthma. (2008) 45:301–8. 10.1080/02770900801911186 18446594

[15] Henderson FW Collier AM Clyde WA Denny FW. Respiratory-syncytial-virus infections, reinfections and immunity. N Engl J Med. (1979) 300:530–4. 10.1056/NEJM197903083001004 763253

[16] Kuh D Ben-Shlomo Y Lynch J Hallqvist J Power C Session 1: Life course epidemiology. J Epidemiol Community Health. (2003) 57:778–83. 10.1136/jech.57.10.778 14573579 PMC1732305

[17] Berendsen ML Smits J Netea MG van der Ven A. Non-specific effects of vaccines and stunting: timing may be essential. EBioMedicine. (2016) 8:341–8. 10.1016/j.ebiom.2016.05.010 27428443 PMC4919612

[18] Brockow I Zutavern A Franke K Schaaf B Berg A Krämer U Einfluss von lebensbedingungen und verhaltensweisen auf die entwicklung von immunsystem und allergien im ost-west-vergleich (LISA) [Influences of lifestyle-related factors on the immune system and the development of allergies in childhood (LISA)]. Monatsschrift Kinderheilkd. (2007) 156:249–55. 10.1007/s00112-007-1527-4

[19] Heinrich J Brüske I Schnappinger M Standl M Flexeder C Thiering E Die zwei deutschen Geburtskohorten GINIplus und LISAplus [Two German Birth Cohorts: GINIplus and LISAplus]. Bundesgesundheitsblatt Gesundheitsforsch Gesundheitsschutz. (2012) 55(6–7):864–74. 10.1007/s00103-012-1485-4 22736169

[20] RKI Epidemiologisches Bulletin 15/97 - impfempfehlung der Ständigen Impfkommission (STIKO) am Robert Koch Institut [Vaccination recommedation of the German standing committee on vaccinations (STIKO)]. Nitag Res. Center. (1997) 13:98–108.

[21] RKI Epidemiologisches Bulletin 15/98 - impfempfehlung der Ständigen Impfkomission (STIKO) am Robert Koch Institut [Vaccination recommedation of the German standing committee on vaccinations (STIKO)]. Nitag Res. Center. (1998) 15:102–14.

[22] Box GEP Cox DR An analysis of transformations. Series B 26 (Methodological). J R Stat Soc. (1964) 26:211–52. 10.1111/j.2517-6161.1964.tb00553.x

[23] Bender R Lange S. Adjusting for multiple testing–when and how? J Clin Epidemiol. (2001) 54:343–9. 10.1016/S0895-4356(00)00314-0 11297884

[24] Nissen TN Birk NM Smits G Jeppesen DL Stensballe LG Netea MG. Bacille Calmette-Guérin (BCG) vaccination at birth and antibody responses to childhood vaccines. A randomised clinical trial. Vaccine. (2017) 35:2084–91. 10.1016/j.vaccine.2017.02.048 28318766

[25] Kleinnijenhuis J van Crevel R Netea MG. Trained immunity: consequences for the heterologous effects of BCG vaccination. Trans R Soc Trop Med Hyg. (2015) 109:29–35. 10.1093/trstmh/tru168 25573107

[26] Selin LK Varga SM Wong IC Welsh RM. Protective heterologous antiviral immunity and enhanced immunopathogenesis mediated by memory T cell populations. J Exp Med. (1998) 188:1705–15. 10.1084/jem.188.9.1705 9802982 PMC2212518

[27] Hollm-Delgado M-G Stuart EA Black RE. Acute lower respiratory infection among Bacille Calmette-Guérin (BCG)–vaccinated children. Pediatrics. (2014) 133:e73–81. 10.1542/peds.2013-2218 24379224

[28] Stensballe LG Nante E Jensen IP Kofoed P-E Poulsen A Jensen H. Acute lower respiratory tract infections and respiratory syncytial virus in infants in Guinea-Bissau: a beneficial effect of BCG vaccination for girls. Vaccine. (2005) 23:1251–7. 10.1016/j.vaccine.2004.09.006 15652667

[29] Bueno SM Gonzalez PA Cautivo KM Mora JE Leiva ED Tobar HE. Protective T cell immunity against respiratory syncytial virus is efficiently induced by recombinant BCG. Proc Natl Acad Sci USA. (2008) 105:20822–7. 10.1073/pnas.0806244105 19075247 PMC2634951

[30] Ritz N Mui M Balloch A Curtis N. Non-specific effect of Bacille Calmette-Guérin vaccine on the immune response to routine immunisations. Vaccine. (2013) 31:3098–103. 10.1016/j.vaccine.2013.03.059 23583897

[31] Welsh RM Selin LK. No one is naive: the significance of heterologous T-cell immunity. Nat Rev Immunol. (2002) 2:417–26. 10.1038/nri820 12093008

[32] Netea MG Quintin J van der Meer JWM. Trained immunity: a memory for innate host defense. Cell Host Microbe. (2011) 9:355–61. 10.1016/j.chom.2011.04.006 21575907

[33] Netea MG van Crevel R. BCG-induced protection: effects on innate immune memory. Semin Immunol. (2014) 26:512–7. 10.1016/j.smim.2014.09.006 25444548

[34] Blok BA Arts RJW van Crevel R Benn CS Netea MG. Trained innate immunity as underlying mechanism for the long-term, nonspecific effects of vaccines. J Leukoc Biol. (2015) 98:347–56. 10.1189/jlb.5RI0315-096R 26150551

[35] Palmeira P Carneiro-Sampaio M. Immunology of breast milk. Rev Assoc Med Bras. (2016) 62:584–93. 10.1590/1806-9282.62.06.584 27849237

[36] Nakagawa S A farewell to Bonferroni: the problems of low statistical power and publication bias. Behav Ecol. (2004) 15:1044–5. 10.1093/beheco/arh107

[37] Camargo A Azuaje F Wang H Zheng H. Permutation - based statistical tests for multiple hypotheses. Source Code Biol Med. (2008) 3:15. 10.1186/1751-0473-3-15 18939983 PMC2611984

