# Supplementary material for: Vaccinations and Infections Are Associated With Unrelated Antibody Titers: An Analysis From the German Birth Cohort Study LISA
Source: Front Pediatr. 2019 Jun 25;7:254. doi: 10.3389/fped.2019.00254 (PMC6603196; doi:10.3389/fped.2019.00254)
Supplement: Supplementary file 1 [file Table_1.DOCX]

**Supplementary Table S1:** Assays with corresponding reference values for detection of seropositive participants for twelve selected IgG titers in the LISA study.

| **IgG titer** | **Assay** | **Cut-off for seropositivity** | **Seropositive**  **% (n)** | **Seronegative**  **% (n)** | **Missing**  **% (n)** |
| --- | --- | --- | --- | --- | --- |
| Measles | Enzygnost Anti-Measles IgG, Dade Behring | > 0.25 U/mL | 67.3 (1,428) | 30.0 (637) | 2.7 (58) |
| Tetanus | Tetanus IgG/Elisa, Sekisui-Virotech | > 0.1 U/mL | 94.3 (2,002) | 5.1 (108) | 0.6 (13) |
| HIB | VaccZyme IgG Haemophilus influenza Typ B Kit, Binding Site | > 1.0 mg/L | 81.1 (1,722) | 18.9 (401) | - |
| CMV | Cobas Core II CMV IgG, Roche | > 0.9 U/mL | 14.0 (298) | 78.4 (1,665) | 7.5 (160) |
| ADV | Adenovirus IgG, Virotech | > 9.0 U/mL | 56.2 (1,194) | 37.8 (803) | 5.9 (126) |
| EBV | Enzygnost Anti-EBV IgG, Dade Behring | > 30.0 U/mL | 14.9 (316) | 79.3 (1,684) | 5.8 (123) |
| HSV | Enzygnost Anti-HSV IgG, Dade Behring | > 100 U/mL | 8.2 (174) | 86 (1,825) | 5.8 (124) |
| HHV-6 | In-house production, infected 9MT4-cells | > 1:10 | 81.2 (1,724) | 12.8 (272) | 6.0 (127) |
| hPIV-3 | PARA-3 Virion/Serion | > 1:10 | 27.4 (582) | 66.2 (1,406) | 6.4 (135) |
| hRSV | In-house production,  Hep-2-cells | > 1:20 | 49.8 (1,057) | 44.4 (943) | 5.8 (123) |
| Influenza A | Fluorimmun Influenza A, Labor Merk | > 1:40 | 30.5 (647) | 63.4 (1,345) | 6.2 (131) |
| Influenza B | Fluorimmun Influenza B, Labor Merk | > 1:40 | 5.4 (115) | 88.4 (1,876) | 6.2 (132) |

ADV, adenovirus; CMV, cytomegalovirus; EBV, Epstein-Barr virus; Hep-2-cells: Human larynx carcinoma; HHV-6, human herpesvirus 6; HIB, *Haemophilus influenzae* type b; hPIV-3, human parainfluenza virus 3; hRSV, human respiratory syncytial virus; HSV, herpes simplex virus; hPIV-3, human parainfluenza virus 3; 9MT4-cells: subclone of HSB-2 (T lymphoblast) cell line persistently infected with HHV-6A
