# Supplementary material for: Vaccinations and Infections Are Associated With Unrelated Antibody Titers: An Analysis From the German Birth Cohort Study LISA
Source: Front Pediatr. 2019 Jun 25;7:254. doi: 10.3389/fped.2019.00254 (PMC6603196; doi:10.3389/fped.2019.00254)
Supplement: Supplementary file 2 [file Table_2.docx]

**Supplementary Table S2:** Adjusted associations between vaccinations, infections, and exposures during pregnancy and IgG titers including missing values of participants in the LISA study*.

| **Antibody titer**^a^ | **Exposure** | **Time^b^** | **Regression coefficient^c^** | **95%-CI** | **p-value** | |
| --- | --- | --- | --- | --- | --- | --- |
| **Measles^d,e^** (n=1,240) |  |  |  |  |  |  |
|  | Chickenpox | 1. | 0.371 | 0.106, 0.636 | 0.006 |  |
|  | Chickenpox | 2. | 0.147 | -0.030, 0.325 | 0.104 |  |
|  | Chickenpox | 1. and 2. | -0.212 | -0.952, 0.527 | 0.573 |  |
|  | Chickenpox | Missing | 0.257 | -0.027, 0.540 | 0.076 |  |
|  | Vaccination against DTPPHIB^f^ | 1. | -0.294 | -0.481, -0.107 | 0.002 |  |
|  | Vaccination against DTPPHIB^f^ | 2. | -0.464 | -2.412, 1.484 | 0.640 |  |
|  | Vaccination against DTPPHIB^f^ | Missing | -0.219 | -0.481, 0.043 | 0.101 |  |
| **Tetanus^d,e^** (n=1,961) |  |  |  |  |  |  |
|  | Vaccination against hepatitis B | 1. | -0.335 | -0.519, -0.151 | <0.001 |  |
|  | Vaccination against hepatitis B | 2. | -0.017 | -0.241, 0.207 | 0.882 |  |
|  | Vaccination against hepatitis B | 1. and 2. | 0.097 | -0.043, 0.236 | 0.173 |  |
|  | Vaccination against hepatitis B | Missing | -0.029 | -0.175, 0.116 | 0.694 |  |
| **HIB^d^**^,^**^e^** (n=1,680) |  |  |  |  |  |  |
|  | Pseudocroup | 1. | -0.015 | -0.319, 0.349 | 0.929 |  |
|  | Pseudocroup | 2. | -0.109 | -0.295, 0.078 | 0.254 |  |
|  | Pseudocroup | 1. and 2. | 0.538 | 0.143, 0.934 | 0.008 |  |
|  | Pseudocroup | Missing | -0.134 | -0.349, 0.081 | 0.222 |  |
|  | Vaccination against hepatitis B | 1. | 0.029 | -0.179, 0.236 | 0.786 |  |
|  | Vaccination against hepatitis B | 2. | 0.279 | 0.0230. 0.529 | 0.028 |  |
|  | Vaccination against hepatitis B | 1. and 2. | 0.215 | 0.056, 0.375 | 0.008 |  |
|  | Vaccination against hepatitis B | Missing | 0.145 | -0.023, 0.312 | 0.091 |  |
| **EBV^e,g^** (n=315) |  |  |  |  |  |  |
|  | Infection^h^ | during pregnancy vs. not | -0.315 | -0.641, -0.011 | 0.058 |  |
|  | Infection^h^ | Missing | -2.094 | -4.093, -0.094 | 0.040 |  |
| **HHV-6** (n=1,705) |  |  |  |  |  |  |
|  | Thrush | 1. | -0.015 | -0.139, 0.109 | 0.815 |  |
|  | Thrush | 2. | 0.244 | 0.084, 0.405 | 0.003 |  |
|  | Thrush | 1. and 2. | -0.059 | -0.231, 0.113 | 0.503 |  |
|  | Thrush | Missing | -0.142 | -0.353, 0.070 | 0.189 |  |
|  | Vaccination^h^ | during pregnancy vs. not | -0.178 | -0.307, -0.049 | 0.007 |  |
|  | Vaccination^h^ | Missing | -0.162 | -1.550, 1.227 | 0.819 |  |
| **hRSV** (n=1,045) |  |  |  |  |  |  |
|  | Vaccination against tuberculosis | 1. | 0.400 | 0.163, 0.637 | 0.001 |  |
|  | Vaccination against tuberculosis | 2. | -0.854 | -1.832, 0.124 | 0.087 |  |
|  | Vaccination against tuberculosis | 1. and 2. | 0.159 | -0.397, 0.714 | 0.575 |  |
|  | Vaccination against tuberculosis | Missing | 0.025 | -0.104, 0.154 | 0.702 |  |
| **Influenza A^g^** (n=643) |  |  |  |  |  |  |
|  | Thrush | 1. | -0.313 | -0.517, -0.110 | 0.003 |  |
|  | Thrush | 2. | -0.225 | -0.491, 0.040 | 0.096 |  |
|  | Thrush | 1. and 2. | -0.168 | -0.457, 0.121 | 0.253 |  |
|  | Thrush | Missing | -0.119 | -0.440, 0.202 | 0.467 |  |
| **ADV^e,g^** (n=1,180) |  |  |  |  |  |  |
|  | Measles^j^ | 1. | -1.754 | -3.130, -0.378 | 0.013 |  |
|  | Measles^j^ | Missing | 0.163 | -0.345, 0.670 | 0.530 |  |

ADV, adenovirus; EBV, Epstein-Barr virus; HIB, Haemophilus influenzae type b; HHV-6, human herpesvirus 6; hRSV, human respiratory syncytial virus. *For one antibody (HSV) the final model had <10 observations in the exposed group; the significant result is not presented.

^a^ Multivariable linear regression, backward selection of independent variables of normalized and standardized IgG antibody titers; only antibody titers were considered with evidence of seropositivity

^b^ 1. =exposure only in first year of age; 2. = exposure only in second year of age; 1. and 2. = exposure only in first two years of age; reference group: children with no corresponding exposure in the first two years of age; Missing = missing values

^c^ Regression coefficients are presented in units of standard deviation of the standard normal distribution; adjusted for sex of child, age of the mother at birth, education level of the parents and study center

^d^ Only IgG titer of vaccinated children; children with corresponding infection were excluded

^e^ In addition adjusted for smoking during pregnancy

^f^ DTPPHIB: vaccination against diphtheria, tetanus, pertussis, HIB and polio; no estimator for “no vaccination” exist, since no measles antibody titer could be detected, accordingly the category “vaccination in both year (1. and 2.)” was selected as reference group

^g^ In addition adjusted for alcohol consumption during pregnancy

^h^ Binary coded

^i^ Only asked in children aged 19 to 24 months
